# Osteomyelitis of Maxilla in Infantile With Periorbital Cellulitis: A Case Report

**DOI:** 10.1097/MD.0000000000001688

**Published:** 2015-10-09

**Authors:** Zhiqiang Feng, Xufeng Chen, Fengdi Cao, Renfa Lai, Qiang Lin

**Affiliations:** From the Department of Stomatology of the First Affiliated Hospital of Jinan University (ZF, RL); and Medicine School of Jinan University, Tianhe District,Guangzhou, China (XC, FC, QL).

## Abstract

Infantile osteomyelitis (IO) is an uncommon and life-threatening disease that can be misdiagnosed. Early diagnosis and treatment can reduce the incidence of sequel.

In this case report, we present a 25-day-old male infant with apparent edema in the entire left periorbital region. Intraorally, the edema occurred in the mucosa of the upper left alveolar region, and 2 draining fistulas with exuded yellow-white pus were present in the left alveolar region. The patient received constant monitoring after admission, and was diagnosed as IO of the maxilla with periorbital cellulitis and sepsis. He also received incision and drainage and anti-inflammatory treatment. After discharge, the patient was followed up for 3 months by phone call, but no recurrence of symptoms was found.

Infantile osteomyelitis is rare in clinic. This case report reminds us of the significance of IO and provides some implications on its diagnosis and treatment.

## INTRODUCTION

Infantile osteomyelitis (IO) is an urgent and serious disease with quick changes in systemic condition. This critical disease is often misdiagnosed and thus requires a selective diagnostic approach.^1^ Apart from the infective etiology, IO is affected by various factors, such as genetic, toxic, and environmental factors.^2^ Its common symptoms include pain, ardent fever, rapid pulse, dysphoria, and even emesia. The complications include ophthalmological changes, airway involvement, infection of cranial cavity, and death.^3,4^ The clinical diagnosis of IO is supported by clinical manifestation and always depends on imaging test. The occurrence of IO is quite rare after the advent of antibiotics. The incidence has been reported at 1/1000 to 3/1000 in neonatal intensive care units,^5^ but IO may still germinate if neglected. Regarding the rarity and diagnostic dilemma of IO, we report here a case of a 25-day-old male infant.

The patient's parents gave written informed consent for this publication.

## CASE REPORT

The 25-day-old male infant was presented to the Outpatient Department of Stomatology with stuffy nose for 1 week, accompanied with red and swollen left eyelid. His mother denied systemic disease or surgical problem of her boy. There was no family history of malignancy.

**FIGURE 1:**
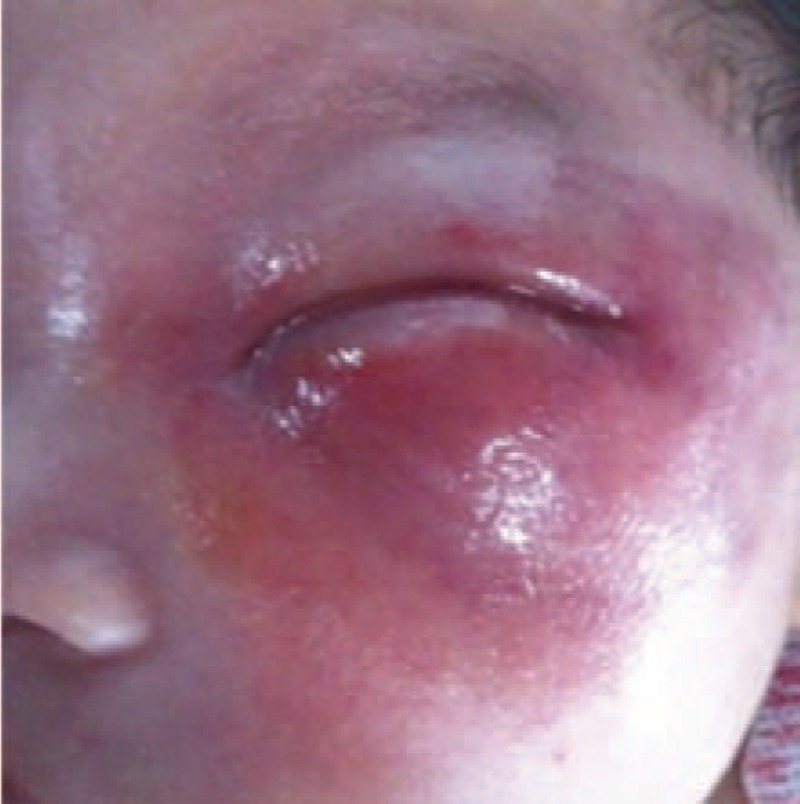
Facial appearance in the first visit. Slight ecstrophy was found in the lower eyelid—conjunctival congestion. The entire left periorbital region was puffy.

**FIGURE 2:**
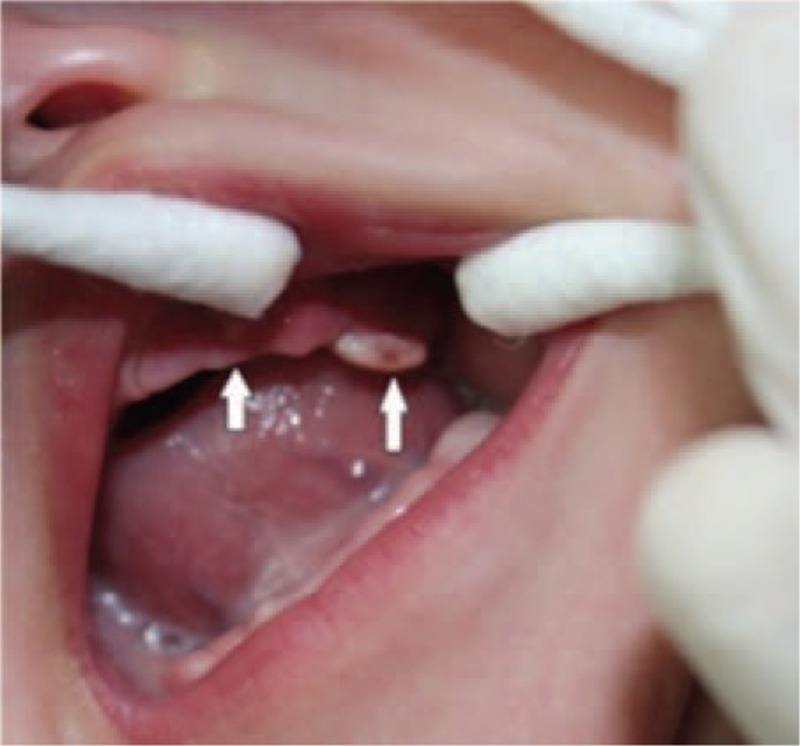
Intraoral, 2 draining fistulas were present in the left alveolar region, with exuded yellow-white pus (indicated by arrows).

**FIGURE 3:**
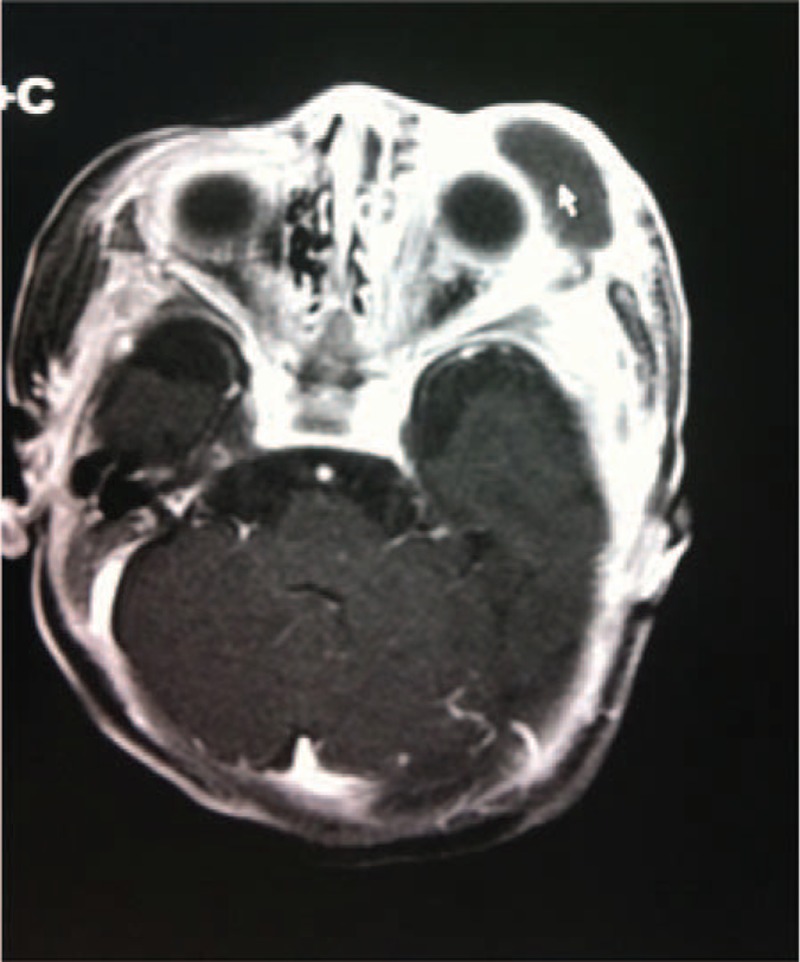
Enhanced MRI showed swelling soft tissue around left orbit, and abscess (indicated by arrows). The signal on bone marrow of external wall of left orbit was intensified. MRI, magnetic resonance imaging.

His vital signs were as follows: temperature: 39.2°C, respiratory rate: 46/min, heart rate: 140/min, and weight: 3.92 kg. Examination revealed nasal obstruction and apparent edema in the left eyelid, and slight ecstrophy in the lower eyelid and conjunctival congestion. The entire left periorbital region was puffy, especially the infraorbital part (Figure 1). Intraorally, 2 draining fistulas as well asedematous mucosa with exuded yellow-white pus were present in the upper left alveolar region (Figure 2). No other positive sign or symptom was found. Laboratory data were: 10.48 × 10^9^/L white blood cell (white blood cell, leucocyte) count, 66.7% neutrophil, 76.6 mg/L C-reactive protein (CRP), and 23.46 g/L calcitonin. Computed tomography (CT) of the head and neck showed that osteomyelitis of the maxilla involved the ethmoidal plate and the left orbit region, and revealed swelling in buccal soft tissues and abscess in the left maxilla. Magnetic resonance imaging (MRI) revealed abscess around the left orbit; inflammation in the left maxillary sinus, ethmoid sinus, and buccal soft tissue; a cone extracellular space in the orbital muscles and extraocular muscles; and secretions in the left meatus nasi (Figure 3). The pathogen was identified by bacterial culture (blood and secretions from the left eye) to be *Staphylococcus aureus*. The patient was diagnosed as IO of the maxilla with periorbital cellulitis and sepsis.

**FIGURE 4:**
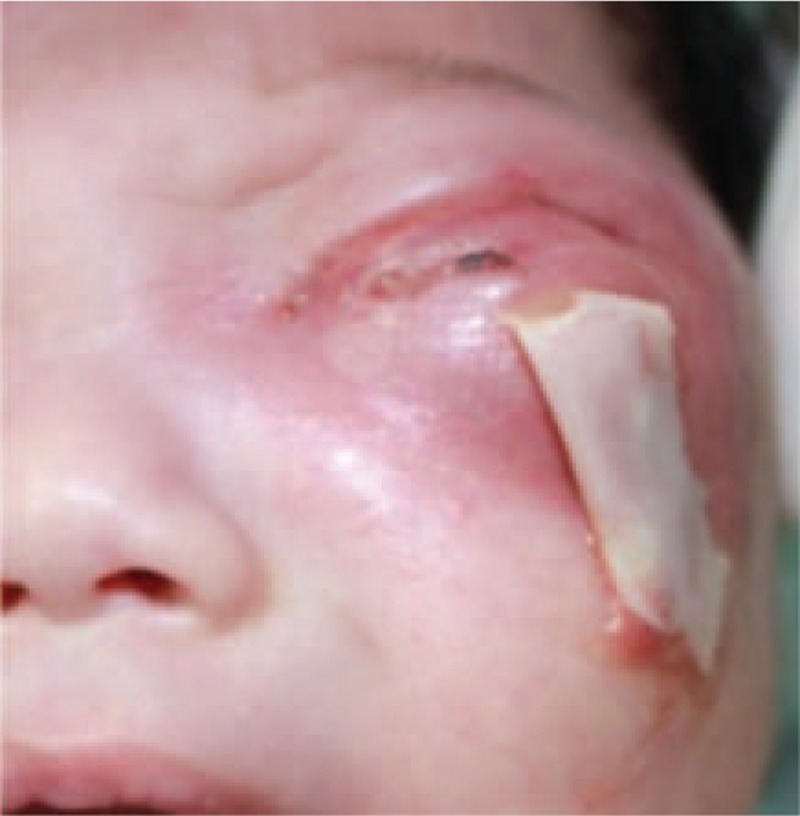
Drain was inserted on the day after I&D. I&D, incision and drainage.

**FIGURE 5:**
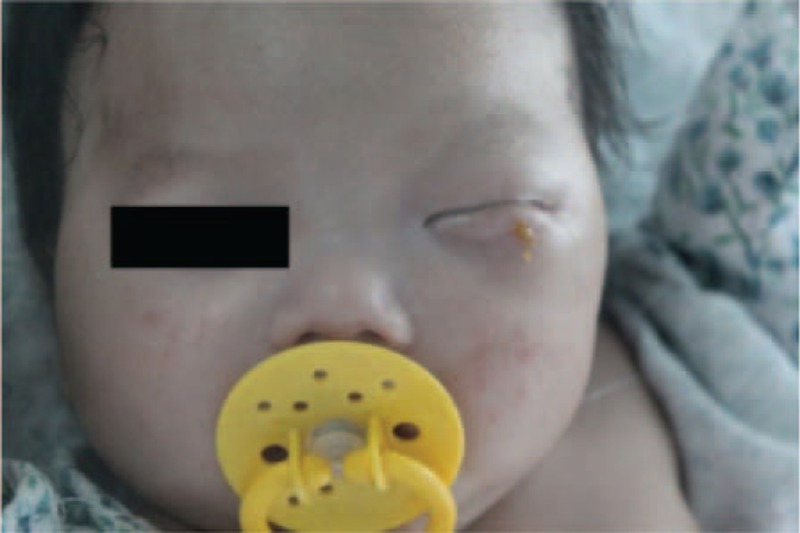
Facial appearance at 1 week after I&D, the periorbital abscess was almost subsided. I&D, incision and drainage.

Constant monitoring was performed after admission. On the basis of the results of bacterial culture and drug-sensitivity test, we prescribed a heavy dose of Cefoperazone and Sulbactam (Sulperazon 0.32 g + 0.9% physiological saline 5 mL/IV guttae (gtt), quaque die (QD)) and Teicoplanin (0.03 g + 0.9% physiological saline 5 mL/IV gtt, QD). Tobramycin eye drops, Levofloxacin eye drops, and Ofloxacin eye ointment were applied alternately every 2 hours (oculus sinister). Compound furacilin nasal drops were diluted 4 times for nasal use. Systemic supportive therapy was performed while oral hygiene was maintained with a chlorhexidine solution. Meanwhile, incision and drainage (I&D) was performed to release the pus and pressure in the left periorbital region, with drain inserted (Figure 4). The fever and laboratory signs of infection regressed and the general condition was stabilized immediately after the operations. The drain was changed and the wound was irrigated with normal saline each day. The drain was removed 3 days later. After 1 week, the periorbital abscess almost subsided, secondary wound was closed, and intraoral fistula healed without any other problem (Figure 5). Anti-inflammatory treatment was performed until recovery and discharge. Three months follow-up after discharge by phone-calling the patient remained symptom-free.

## DISCUSSION

The first case of IO which occurred in jaws was reported in 1932.^6^ In most cases since then, IO resulted from the hematogenic spread of bacteria. IO could be triggered by traumatic injuries with open wounds in the patient's oral mucosa after bacterial invasion, or by the suppurative mastitis in the infant's mother. IO is sometimes associated with dacryocystitis or nasolacrimal duct inflammation.^7,8^ It may occur secondary to malignant osteopetrosis, but rarely.^9,10^ In our case, the patient's mother denied history of mastitis, systemic disease, or surgical problem of her boy. Combining the physical examination with medical history, we believed the bacteria that invaded the patient's damaged oral mucosa came from his mother's contaminated nipples. These factors caused local infection and then induced acute suppurative osteomyelitis. After the infection ascended to the maxillary region, inflammation progressed to be periorbital abscess, which penetrated through the cortical bone.

Commonly, the pathogen of IO is *S aureus*,^11^ and the first-choice antibiotics are penicillin and cephalosporin. In case of abscess, the types of antibiotics can be adjusted depending on the results of the bacterial culture (from pus) and drug-sensitivity test. When the clinical symptoms are improved, antibacterial treatment should be used for another 1 to 2 weeks to prevent the recurrence of inflammation.^12^ Termination of antibiotic administration could be considered if hypersensitive CRP (hs-CRP) level—the index monitoring the course of disease—is <20 mg/L.^13^ Curettage, which would damage the tooth germ and bones, was not used during I&D. In this case, we did not widen the fistula that had existed in the alveolar region. We wondered whether the pus flowing out from the intraoral fistula might cause the complications, such as lung abscess and disseminated intestinal abscess, if the pus was aspirated into the patient's lung. On the contrary, regarding the obvious periorbital abscess, we decided to perform I&D to the periorbital area. The combination of I&D and systemic antibiotic therapy led to inflammation regression and effectively avoided the worsening of osteomyelitis.

## CONCLUSIONS

Recognition of this entity is important, since IO is life-threatening and can be often misdiagnosed. Diagnosis of IO can be realized through clinical manifestation and imaging tests. In case of misdiagnosis, routine X-ray or CT scans are necessary when edema or fistula occurs in the periorbital, maxillofacial, or intraoral region of the infant. Treatments of IO include early diagnosis, I&D (in case of abscess formation), bacterial culture, and drug-sensitivity test with appropriate antibiotic therapy, and supportive therapy. Surgical treatment is available if needed. Early diagnosis and treatment can reduce the incidence of sequel. In this case, the combination of antibiotic therapy and I&D is effective without any complication during the 3 months follow-up. This case report reminds us of the significance of IO and provides some implications for its diagnosis and treatment.
